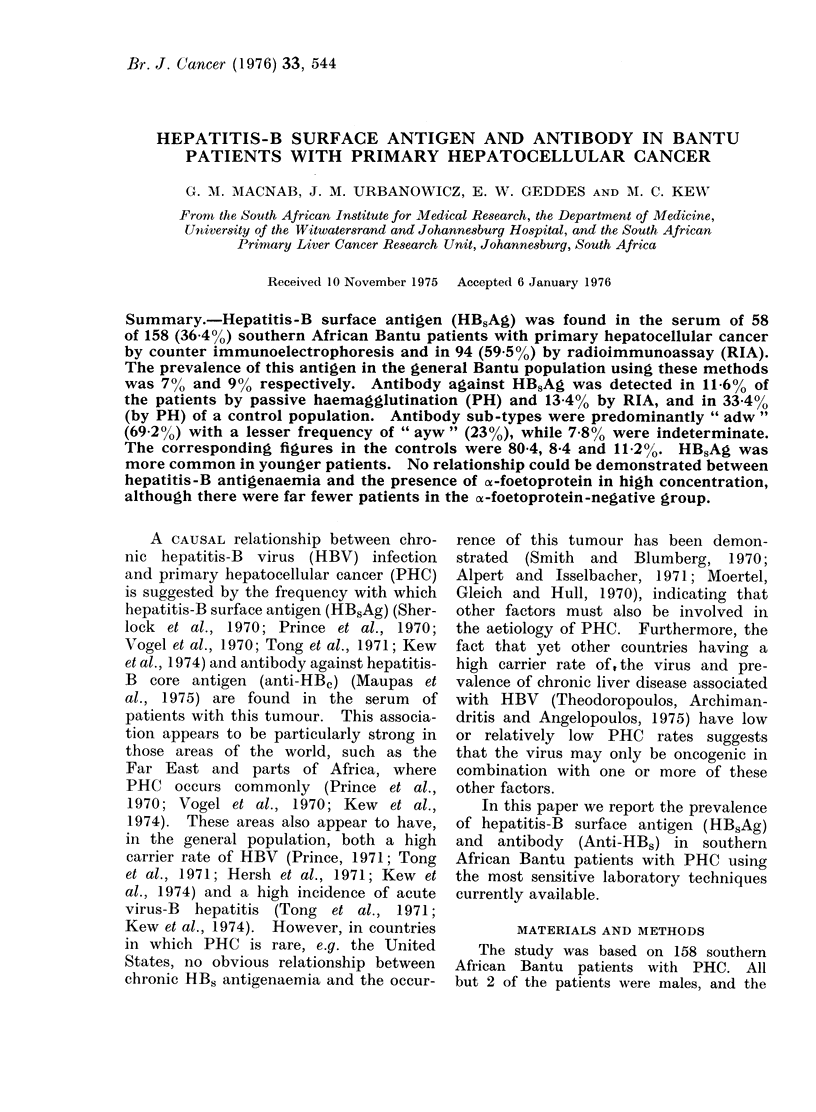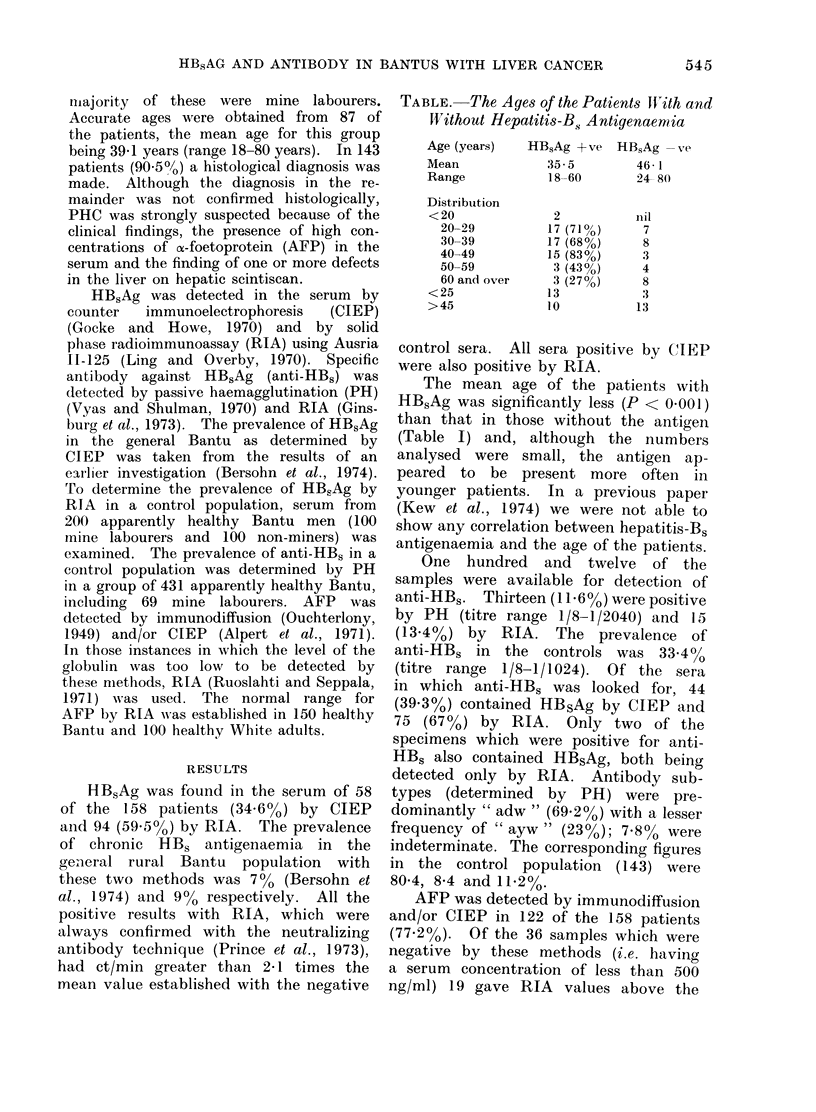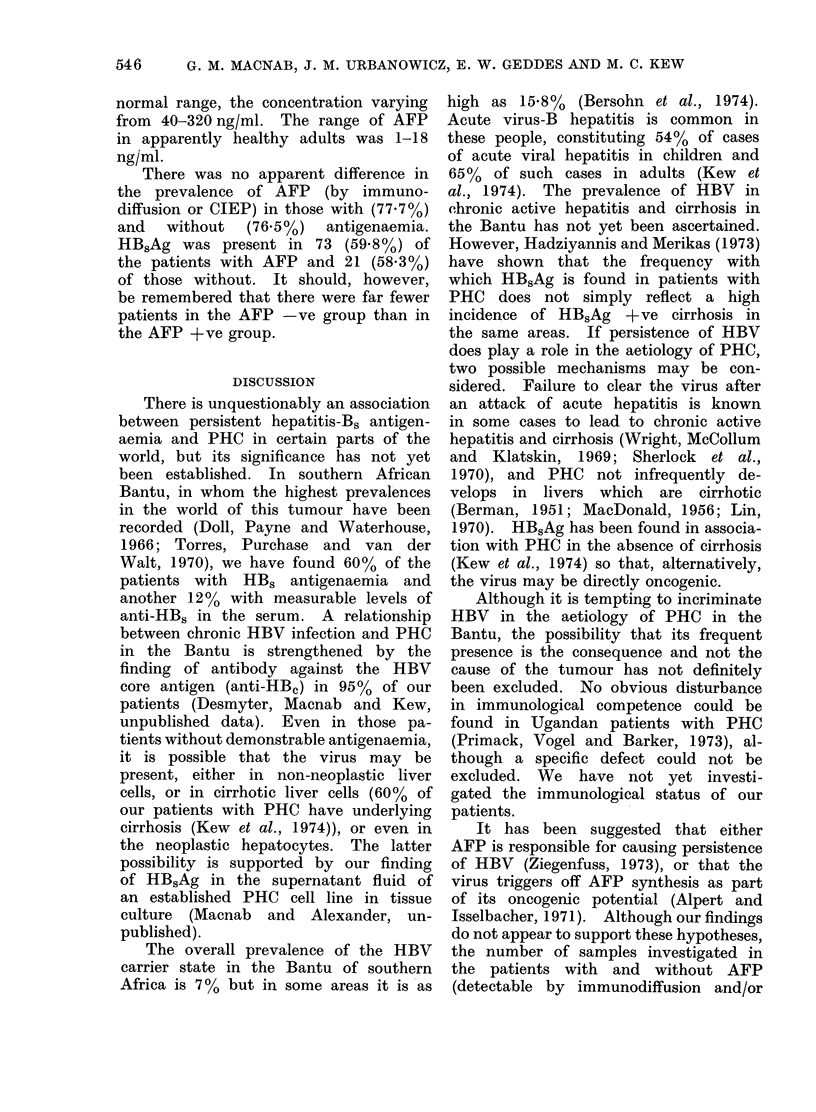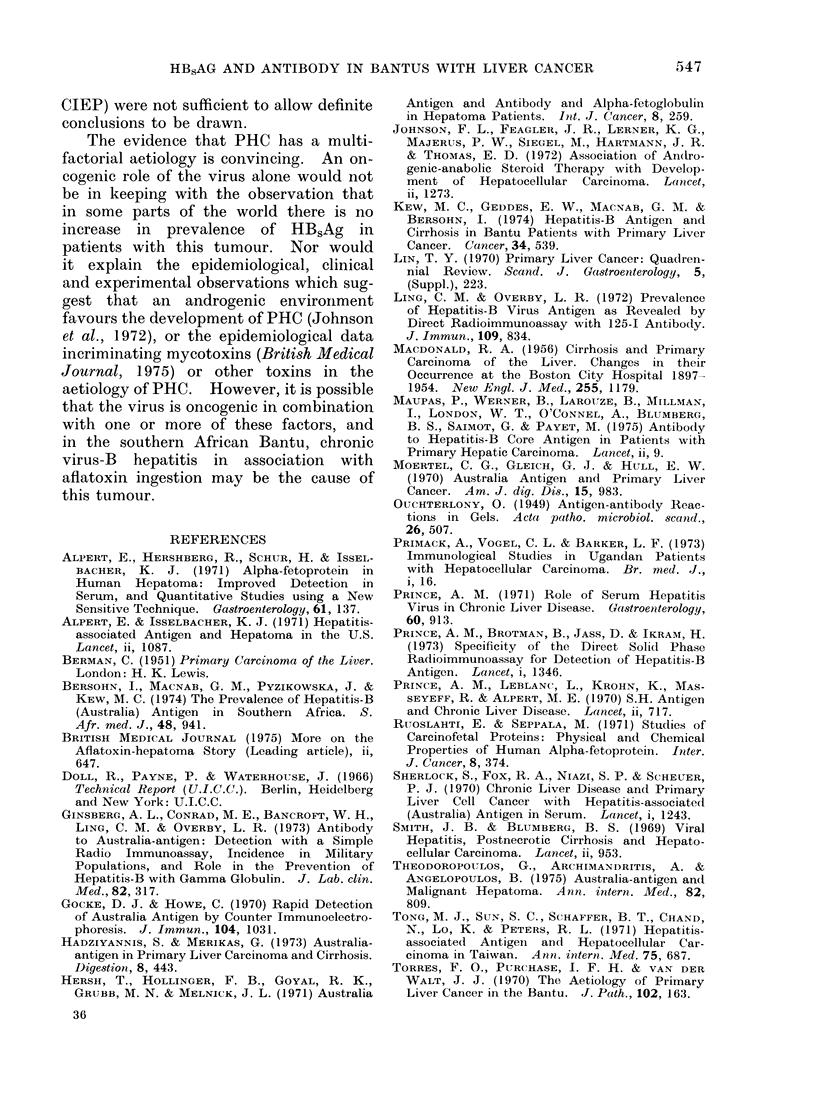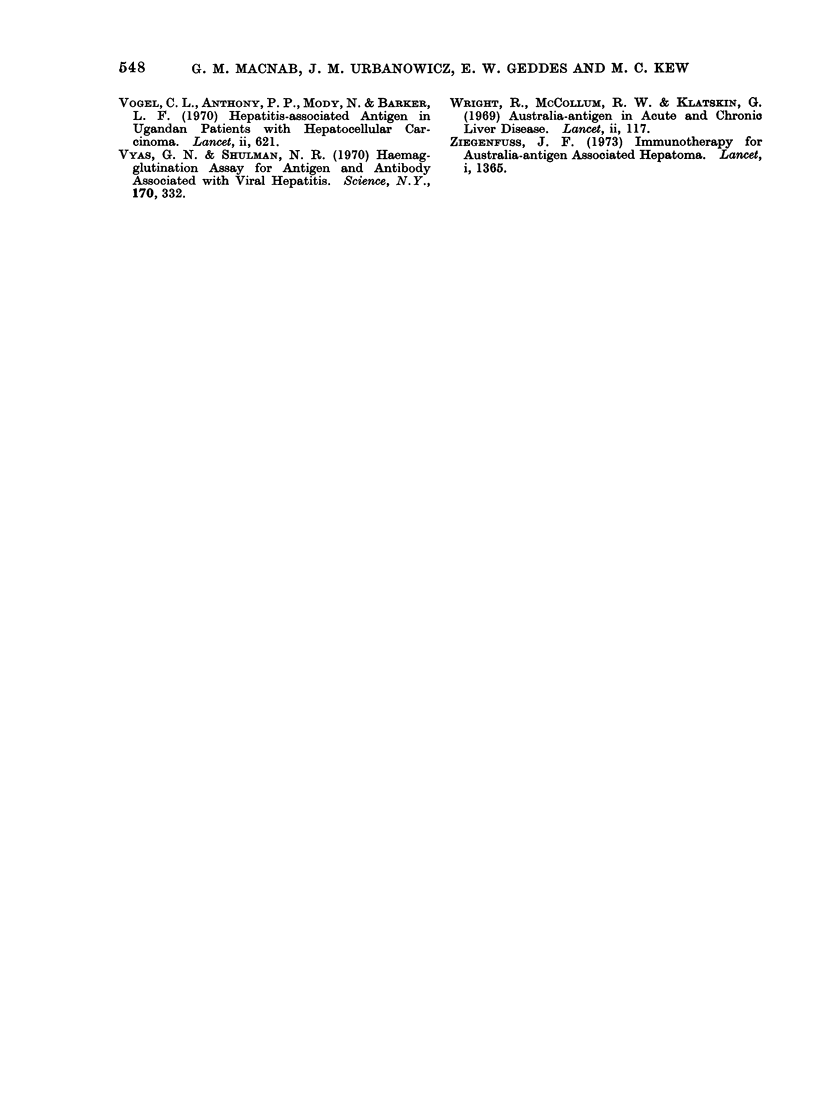# Hepatitis-B surface antigen and antibody in Bantu patients with primary hepatocellular cancer.

**DOI:** 10.1038/bjc.1976.86

**Published:** 1976-05

**Authors:** G. M. Macnab, J. M. Urbanowicz, E. W. Geddes, M. C. Kew

## Abstract

Hepatitis-B surface antigen (HBsAg) was found in the serum of 58 of 158 (36-4%) southern African Bantu patients with primary hepatocellular cancer by counter immunoelectrophoresis and in 94 (59-5%) by radioimmunoassay (RIA). The prevalence of this antigen in the general Bantu population using these methods was 7% and 9% respectively. Antibody against HBsAg was detected in 11-6% of the patients by passive haemagglutination (PH) and 13-4% by RIA, and in 33-4% (by PH) of a control population. Antibody sub-types were predominantly "adw" (69-2%) with a lesser frequency of "ayw" (23%), while 7-8% were indeterminate. The corresponding figures in the controls were 80-4, 8-4 and 11-2%. HBsAg was more common in younger patients. No relationship could be demonstrated between hepatitis-B antigenaemia and the presence of alpha-foetoprotein in high concentration, although there were far fewer patients in the alpha-foetoprotein-negative group.


*Br. J. Cancer* (1976) **33**, 544

### HEPATITIS-B SURFACE ANTIGEN AND ANTIBODY IN BANTU PATIENTS WITH PRIMARY HEPATOCELLULAR CANCER

G. M. MACNAB, J. M. URBANOWICZ, E. W. GEDDES AND M. C. KEW

*From the South African Institute for Medical Research, the Department of Medicine, University of the Witwatersrand and Johannesburg Hospital, and the South African Primary Liver Cancer Research Unit, Johannesburg, South Africa*

Received 10 November 1975 Accepted 6 January 1976

**Summary.—Hepatitis-B surface antigen ($HB_sAg$) was found in the serum of 58 of 158 (36·4%) southern African Bantu patients with primary hepatocellular cancer by counter immunoelectrophoresis and in 94 (59·5%) by radioimmunoassay (RIA). The prevalence of this antigen in the general Bantu population using these methods was 7% and 9% respectively. Antibody against $HB_sAg$ was detected in 11·6% of the patients by passive haemagglutination (PH) and 13·4% by RIA, and in 33·4% (by PH) of a control population. Antibody sub-types were predominantly "adw" (69·2%) with a lesser frequency of "ayw" (23%), while 7·8% were indeterminate. The corresponding figures in the controls were 80·4, 8·4 and 11·2%. $HB_sAg$ was more common in younger patients. No relationship could be demonstrated between hepatitis-B antigenaemia and the presence of α-foetoprotein in high concentration, although there were far fewer patients in the α-foetoprotein-negative group.**

A CAUSAL relationship between chronic hepatitis-B virus (HBV) infection and primary hepatocellular cancer (PHC) is suggested by the frequency with which hepatitis-B surface antigen ($HB_sAg$) (Sherlock *et al.*, 1970; Prince *et al.*, 1970; Vogel *et al.*, 1970; Tong *et al.*, 1971; Kew *et al.*, 1974) and antibody against hepatitis-B core antigen (anti-$HB_c$) (Maupas *et al.*, 1975) are found in the serum of patients with this tumour. This association appears to be particularly strong in those areas of the world, such as the Far East and parts of Africa, where PHC occurs commonly (Prince *et al.*, 1970; Vogel *et al.*, 1970; Kew *et al.*, 1974). These areas also appear to have, in the general population, both a high carrier rate of HBV (Prince, 1971; Tong *et al.*, 1971; Hersh *et al.*, 1971; Kew *et al.*, 1974) and a high incidence of acute virus-B hepatitis (Tong *et al.*, 1971; Kew *et al.*, 1974). However, in countries in which PHC is rare, *e.g.* the United States, no obvious relationship between chronic $HB_s$ antigenaemia and the occurrence of this tumour has been demonstrated (Smith and Blumberg, 1970; Alpert and Isselbacher, 1971; Moertel, Gleich and Hull, 1970), indicating that other factors must also be involved in the aetiology of PHC. Furthermore, the fact that yet other countries having a high carrier rate of the virus and prevalence of chronic liver disease associated with HBV (Theodoropoulos, Archimandritis and Angelopoulos, 1975) have low or relatively low PHC rates suggests that the virus may only be oncogenic in combination with one or more of these other factors.

In this paper we report the prevalence of hepatitis-B surface antigen ($HB_sAg$) and antibody (Anti-$HB_s$) in southern African Bantu patients with PHC using the most sensitive laboratory techniques currently available.

### MATERIALS AND METHODS

The study was based on 158 southern African Bantu patients with PHC. All but 2 of the patients were males, and the majority of these were mine labourers. Accurate ages were obtained from 87 of the patients, the mean age for this group being 39·1 years (range 18–80 years). In 143 patients (90·5%) a histological diagnosis was made. Although the diagnosis in the remainder was not confirmed histologically, PHC was strongly suspected because of the clinical findings, the presence of high concentrations of $\alpha$-foetoprotein (AFP) in the serum and the finding of one or more defects in the liver on hepatic scintiscan.

$HB_sAg$ was detected in the serum by counter immunoelectrophoresis (CIEP) (Gocke and Howe, 1970) and by solid phase radioimmunoassay (RIA) using Ausria II-125 (Ling and Overby, 1970). Specific antibody against $HB_sAg$ (anti-$HB_s$) was detected by passive haemagglutination (PH) (Vyas and Shulman, 1970) and RIA (Ginsburg *et al.*, 1973). The prevalence of $HB_sAg$ in the general Bantu as determined by CIEP was taken from the results of an earlier investigation (Bersohn *et al.*, 1974). To determine the prevalence of $HB_sAg$ by RIA in a control population, serum from 200 apparently healthy Bantu men (100 mine labourers and 100 non-miners) was examined. The prevalence of anti-$HB_s$ in a control population was determined by PH in a group of 431 apparently healthy Bantu, including 69 mine labourers. AFP was detected by immunodiffusion (Ouchterlony, 1949) and/or CIEP (Alpert *et al.*, 1971). In those instances in which the level of the globulin was too low to be detected by these methods, RIA (Ruoslahti and Seppala, 1971) was used. The normal range for AFP by RIA was established in 150 healthy Bantu and 100 healthy White adults.

### RESULTS

$HB_sAg$ was found in the serum of 58 of the 158 patients (34·6%) by CIEP and 94 (59·5%) by RIA. The prevalence of chronic $HB_s$ antigenaemia in the general rural Bantu population with these two methods was 7% (Bersohn *et al.*, 1974) and 9% respectively. All the positive results with RIA, which were always confirmed with the neutralizing antibody technique (Prince *et al.*, 1973), had ct/min greater than 2·1 times the mean value established with the negative control sera. All sera positive by CIEP were also positive by RIA.

TABLE.—*The Ages of the Patients With and Without Hepatitis-$B_s$ Antigenaemia*

| Age (years) | $HB_sAg$ +ve | $HB_sAg$ −ve |
|---|---|---|
| Mean | 35·5 | 46·1 |
| Range | 18–60 | 24–80 |
| Distribution | | |
| <20 | 2 | nil |
| 20–29 | 17 (71%) | 7 |
| 30–39 | 17 (68%) | 8 |
| 40–49 | 15 (83%) | 3 |
| 50–59 | 3 (43%) | 4 |
| 60 and over | 3 (27%) | 8 |
| <25 | 13 | 3 |
| >45 | 10 | 13 |

The mean age of the patients with $HB_sAg$ was significantly less ($P < 0·001$) than that in those without the antigen (Table I) and, although the numbers analysed were small, the antigen appeared to be present more often in younger patients. In a previous paper (Kew *et al.*, 1974) we were not able to show any correlation between hepatitis-$B_s$ antigenaemia and the age of the patients.

One hundred and twelve of the samples were available for detection of anti-$HB_s$. Thirteen (11·6%) were positive by PH (titre range 1/8–1/2040) and 15 (13·4%) by RIA. The prevalence of anti-$HB_s$ in the controls was 33·4% (titre range 1/8–1/1024). Of the sera in which anti-$HB_s$ was looked for, 44 (39·3%) contained $HB_sAg$ by CIEP and 75 (67%) by RIA. Only two of the specimens which were positive for anti-$HB_s$ also contained $HB_sAg$, both being detected only by RIA. Antibody subtypes (determined by PH) were predominantly " adw " (69·2%) with a lesser frequency of " ayw " (23%); 7·8% were indeterminate. The corresponding figures in the control population (143) were 80·4, 8·4 and 11·2%.

AFP was detected by immunodiffusion and/or CIEP in 122 of the 158 patients (77·2%). Of the 36 samples which were negative by these methods (*i.e.* having a serum concentration of less than 500 ng/ml) 19 gave RIA values above the normal range, the concentration varying from 40–320 ng/ml. The range of AFP in apparently healthy adults was 1–18 ng/ml.

There was no apparent difference in the prevalence of AFP (by immunodiffusion or CIEP) in those with (77·7%) and without (76·5%) antigenaemia. $HB_sAg$ was present in 73 (59·8%) of the patients with AFP and 21 (58·3%) of those without. It should, however, be remembered that there were far fewer patients in the AFP −ve group than in the AFP +ve group.

### DISCUSSION

There is unquestionably an association between persistent hepatitis-$B_s$ antigenaemia and PHC in certain parts of the world, but its significance has not yet been established. In southern African Bantu, in whom the highest prevalences in the world of this tumour have been recorded (Doll, Payne and Waterhouse, 1966; Torres, Purchase and van der Walt, 1970), we have found 60% of the patients with $HB_s$ antigenaemia and another 12% with measurable levels of anti-$HB_s$ in the serum. A relationship between chronic HBV infection and PHC in the Bantu is strengthened by the finding of antibody against the HBV core antigen (anti-$HB_c$) in 95% of our patients (Desmyter, Macnab and Kew, unpublished data). Even in those patients without demonstrable antigenaemia, it is possible that the virus may be present, either in non-neoplastic liver cells, or in cirrhotic liver cells (60% of our patients with PHC have underlying cirrhosis (Kew *et al.*, 1974)), or even in the neoplastic hepatocytes. The latter possibility is supported by our finding of $HB_sAg$ in the supernatant fluid of an established PHC cell line in tissue culture (Macnab and Alexander, unpublished).

The overall prevalence of the HBV carrier state in the Bantu of southern Africa is 7% but in some areas it is as high as 15·8% (Bersohn *et al.*, 1974). Acute virus-B hepatitis is common in these people, constituting 54% of cases of acute viral hepatitis in children and 65% of such cases in adults (Kew *et al.*, 1974). The prevalence of HBV in chronic active hepatitis and cirrhosis in the Bantu has not yet been ascertained. However, Hadziyannis and Merikas (1973) have shown that the frequency with which $HB_sAg$ is found in patients with PHC does not simply reflect a high incidence of $HB_sAg$ +ve cirrhosis in the same areas. If persistence of HBV does play a role in the aetiology of PHC, two possible mechanisms may be considered. Failure to clear the virus after an attack of acute hepatitis is known in some cases to lead to chronic active hepatitis and cirrhosis (Wright, McCollum and Klatskin, 1969; Sherlock *et al.*, 1970), and PHC not infrequently develops in livers which are cirrhotic (Berman, 1951; MacDonald, 1956; Lin, 1970). $HB_sAg$ has been found in association with PHC in the absence of cirrhosis (Kew *et al.*, 1974) so that, alternatively, the virus may be directly oncogenic.

Although it is tempting to incriminate HBV in the aetiology of PHC in the Bantu, the possibility that its frequent presence is the consequence and not the cause of the tumour has not definitely been excluded. No obvious disturbance in immunological competence could be found in Ugandan patients with PHC (Primack, Vogel and Barker, 1973), although a specific defect could not be excluded. We have not yet investigated the immunological status of our patients.

It has been suggested that either AFP is responsible for causing persistence of HBV (Ziegenfuss, 1973), or that the virus triggers off AFP synthesis as part of its oncogenic potential (Alpert and Isselbacher, 1971). Although our findings do not appear to support these hypotheses, the number of samples investigated in the patients with and without AFP (detectable by immunodiffusion and/or

CIEP) were not sufficient to allow definite conclusions to be drawn.

The evidence that PHC has a multifactorial aetiology is convincing. An oncogenic role of the virus alone would not be in keeping with the observation that in some parts of the world there is no increase in prevalence of HB$_S$Ag in patients with this tumour. Nor would it explain the epidemiological, clinical and experimental observations which suggest that an androgenic environment favours the development of PHC (Johnson *et al.*, 1972), or the epidemiological data incriminating mycotoxins (*British Medical Journal*, 1975) or other toxins in the aetiology of PHC. However, it is possible that the virus is oncogenic in combination with one or more of these factors, and in the southern African Bantu, chronic virus-B hepatitis in association with aflatoxin ingestion may be the cause of this tumour.

### REFERENCES

ALPERT, E., HERSHBERG, R., SCHUR, H. & ISSELBACHER, K. J. (1971) Alpha-fetoprotein in Human Hepatoma: Improved Detection in Serum, and Quantitative Studies using a New Sensitive Technique. *Gastroenterology*, **61**, 137.

ALPERT, E. & ISSELBACHER, K. J. (1971) Hepatitis-associated Antigen and Hepatoma in the U.S. *Lancet*, ii, 1087.

BERMAN, C. (1951) *Primary Carcinoma of the Liver.* London: H. K. Lewis.

BERSOHN, I., MACNAB, G. M., PYZIKOWSKA, J. & KEW, M. C. (1974) The Prevalence of Hepatitis-B (Australia) Antigen in Southern Africa. *S. Afr. med. J.*, **48**, 941.

BRITISH MEDICAL JOURNAL (1975) More on the Aflatoxin-hepatoma Story (Leading article), ii, 647.

DOLL, R., PAYNE, P. & WATERHOUSE, J. (1966) *Technical Report* (*U.I.C.C.*). Berlin, Heidelberg and New York: U.I.C.C.

GINSBERG, A. L., CONRAD, M. E., BANCROFT, W. H., LING, C. M. & OVERBY, L. R. (1973) Antibody to Australia-antigen: Detection with a Simple Radio Immunoassay, Incidence in Military Populations, and Role in the Prevention of Hepatitis-B with Gamma Globulin. *J. Lab. clin. Med.*, **82**, 317.

GOCKE, D. J. & HOWE, C. (1970) Rapid Detection of Australia Antigen by Counter Immunoelectrophoresis. *J. Immun.*, **104**, 1031.

HADZIYANNIS, S. & MERIKAS, G. (1973) Australia-antigen in Primary Liver Carcinoma and Cirrhosis. *Digestion*, **8**, 443.

HERSH, T., HOLLINGER, F. B., GOYAL, R. K., GRUBB, M. N. & MELNICK, J. L. (1971) Australia Antigen and Antibody and Alpha-fetoglobulin in Hepatoma Patients. *Int. J. Cancer*, **8**, 259.

JOHNSON, F. L., FEAGLER, J. R., LERNER, K. G., MAJERUS, P. W., SIEGEL, M., HARTMANN, J. R. & THOMAS, E. D. (1972) Association of Androgenic-anabolic Steroid Therapy with Development of Hepatocellular Carcinoma. *Lancet*, ii, 1273.

KEW, M. C., GEDDES, E. W., MACNAB, G. M. & BERSOHN, I. (1974) Hepatitis-B Antigen and Cirrhosis in Bantu Patients with Primary Liver Cancer. *Cancer*, **34**, 539.

LIN, T. Y. (1970) Primary Liver Cancer: Quadrennial Review. *Scand. J. Gastroenterology*, **5**, (Suppl.), 223.

LING, C. M. & OVERBY, L. R. (1972) Prevalence of Hepatitis-B Virus Antigen as Revealed by Direct Radioimmunoassay with 125-I Antibody. *J. Immun.*, **109**, 834.

MACDONALD, R. A. (1956) Cirrhosis and Primary Carcinoma of the Liver. Changes in their Occurrence at the Boston City Hospital 1897–1954. *New Engl. J. Med.*, **255**, 1179.

MAUPAS, P., WERNER, B., LAROUZE, B., MILLMAN, I., LONDON, W. T., O'CONNEL, A., BLUMBERG, B. S., SAIMOT, G. & PAYET, M. (1975) Antibody to Hepatitis-B Core Antigen in Patients with Primary Hepatic Carcinoma. *Lancet*, ii, 9.

MOERTEL, C. G., GLEICH, G. J. & HULL, E. W. (1970) Australia Antigen and Primary Liver Cancer. *Am. J. dig. Dis.*, **15**, 983.

OUCHTERLONY, O. (1949) Antigen-antibody Reactions in Gels. *Acta patho. microbiol. scand.*, **26**, 507.

PRIMACK, A., VOGEL, C. L. & BARKER, L. F. (1973) Immunological Studies in Ugandan Patients with Hepatocellular Carcinoma. *Br. med. J.*, i, 16.

PRINCE, A. M. (1971) Role of Serum Hepatitis Virus in Chronic Liver Disease. *Gastroenterology*, **60**, 913.

PRINCE, A. M., BROTMAN, B., JASS, D. & IKRAM, H. (1973) Specificity of the Direct Solid Phase Radioimmunoassay for Detection of Hepatitis-B Antigen. *Lancet*, i, 1346.

PRINCE, A. M., LEBLANC, L., KROHN, K., MASSEYEFF, R. & ALPERT, M. E. (1970) S.H. Antigen and Chronic Liver Disease. *Lancet*, ii, 717.

RUOSLAHTI, E. & SEPPALA, M. (1971) Studies of Carcinofetal Proteins: Physical and Chemical Properties of Human Alpha-fetoprotein. *Inter. J. Cancer*, **8**, 374.

SHERLOCK, S., FOX, R. A., NIAZI, S. P. & SCHEUER, P. J. (1970) Chronic Liver Disease and Primary Liver Cell Cancer with Hepatitis-associated (Australia) Antigen in Serum. *Lancet*, i, 1243.

SMITH, J. B. & BLUMBERG, B. S. (1969) Viral Hepatitis, Postnecrotic Cirrhosis and Hepatocellular Carcinoma. *Lancet*, ii, 953.

THEODOROPOULOS, G., ARCHIMANDRITIS, A. & ANGELOPOULOS, B. (1975) Australia-antigen and Malignant Hepatoma. *Ann. intern. Med.*, **82**, 809.

TONG, M. J., SUN, S. C., SCHAFFER, B. T., CHAND, N., LO, K. & PETERS, R. L. (1971) Hepatitis-associated Antigen and Hepatocellular Carcinoma in Taiwan. *Ann. intern. Med.* **75**, 687.

TORRES, F. O., PURCHASE, I. F. H. & VAN DER WALT, J. J. (1970) The Aetiology of Primary Liver Cancer in the Bantu. *J. Path.*, **102**, 163.

VOGEL, C. L., ANTHONY, P. P., MODY, N. & BARKER, L. F. (1970) Hepatitis-associated Antigen in Ugandan Patients with Hepatocellular Carcinoma. *Lancet*, ii, 621.

VYAS, G. N. & SHULMAN, N. R. (1970) Haemagglutination Assay for Antigen and Antibody Associated with Viral Hepatitis. *Science, N.Y.*, **170**, 332.

WRIGHT, R., MCCOLLUM, R. W. & KLATSKIN, G. (1969) Australia-antigen in Acute and Chronic Liver Disease. *Lancet*, ii, 117.

ZIEGENFUSS, J. F. (1973) Immunotherapy for Australia-antigen Associated Hepatoma. *Lancet*, i, 1365.